# Impact of authentic leadership on employee turnover intention: Perceived supervisor support as mediator and organizational identification as moderator

**DOI:** 10.3389/fpsyg.2023.1009639

**Published:** 2023-01-24

**Authors:** Kiho Jun, Zhehua Hu, Yi Sun

**Affiliations:** BNU-HKBU United International College, Zhuhai, China

**Keywords:** authentic leadership, turnover intention, perceived supervisor support, organizational identification, leadership

## Abstract

Authentic leadership is considered a critical factor for retaining talented employees. However, despite fruitful findings, researchers have paid little attention to how authentic leadership is associated with employee turnover intention. Drawing on organizational support theory, justice literature, and social identity theory, we examine the effects of supervisors’ authentic leadership on employee turnover intention to better understand how authentic leaders reduce employees’ turnover intention in Asian context. In this study, we focus on the mediating role of perceived supervisor support (PSS) and the moderating role of organizational identification in the relationship between supervisors’ authentic leadership and employee turnover intention. To test our hypothesized research model, we adopted a cross-sectional design with a convenience data sampling. We also used a self-report research design in the current study. We collected data from 433 employees from several organizations in Korea. Our respondents rated their immediate supervisors’ authentic leadership and their PSS, turnover intention and organizational identification. Confirmatory factor analysis, regression analysis, and moderated mediation analysis revealed that: authentic leadership negatively predicted employee turnover intention. In addition, PSS completely mediates the relationship between authentic leadership and employee turnover intention. Furthermore, organizational identification moderates the relationship between PSS and turnover intention. Lastly, organizational identification moderates the mediating effect of PSS on the relationships between perceptions of authentic leadership and employee turnover intention. Herein, we discuss the managerial implications and future research directions arising from our study.

## Introduction

The novel coronavirus (COVID-19) outbreak has severely impacted the world for more than 2 years. According to ILO-OECD (2020), the pandemic is causing massive damage to the world economy, resulting in large-scale furloughs and layoffs. The employment relationship is characterized by higher uncertainty now than before the pandemic, resulting in high-level job insecurity and anxiety among employees (Trougakos et al., 2020). Therefore, the unstable employment relationship might lead to high turnover rate in any organization during or after pandemic. In the past decades, organizational scholars have noted that employee turnover might be a serious problem to organizational functioning because a high turnover rate has negative implications for several dimensions of organizational performance (e.g., safety, productivity, and monetary; Holtom et al., 2008; Shaw, 2011). Turnover has been explained using several psychological concepts, such as organizational commitment, justice perceptions, and burnout (Holtom et al., 2008). Turnover research has also incorporated an increasing number of variables that consider employees’ relationships with their environment (e.g., with the organization, supervisor, and co-workers). For example, the establishment of mentoring relationships was shown to reduce protégés’ turnover intentions (Payne and Huffman, 2005). In addition, hindrance stressors (e.g., organizational politics, hassle, situational constraints, role conflict, and role overload) have been found to lead to higher turnover intentions (Podsakoff et al., 2007). Leaders who frequently interact with followers and thus significantly influence their behaviors and attitudes are also expected to impact employee turnover intention substantially. For example, leadership behaviors such as ethical leadership (Brown et al., 2005), transformational leadership (Wang et al., 2011), paternalistic leadership (Bedi, 2020), and authentic leadership (Lemoine et al., 2019) are strongly associated with several outcomes including turnover and turnover intention. Moreover, high leader–member exchange (LMX) reduces employee turnover intention and actual turnover by facilitating relationship development between leaders and followers (Lord et al., 2017; Mumtaz and Rowley, 2020).

Given that employee turnover is directly influenced by employees’ relationships with their supervisors (Lord et al., 2017; Mumtaz and Rowley, 2020), we argue that authentic leadership might be effective at retaining key employees through the development of trust and authentic relationships (Avolio et al., 2004). Over the past decades, publicized scandals and leadership failures turned the leadership research focus to an internal moral perspective, in which authentic leadership has emerged as a leadership style that complements the work on ethical and transformational leadership (George, 2003; Avolio et al., 2004; Gardner et al., 2005; Ilies et al., 2005). Indeed, Avolio and Gardner (2005) argued that authentic leadership is the root construct of positive forms of leadership—a basis of multiple leadership styles and behaviors, such as ethical or transformational leadership. Researchers have empirically studied diverse issues associated with the effects of authentic leadership on employees and organizations. Recent reviews have shown that authentic leadership positively affects employee attitudes and behaviors, such as work engagement, organizational citizenship behavior (OCB), and performance (Gardner et al., 2011; Lemoine et al., 2019). Recently, research on the relationship between turnover and authentic leadership has received significant attention. With an increased theoretical emphasis on the role of authentic leadership in retaining employees (Avolio et al., 2004), thus, the effects of authentic leadership on employee turnover intention have been examined (e.g., Ausar et al., 2016). Scholars have examined how authentic leadership influences employee turnover intention. For example, scholars have found that employees’ work engagement mediates the direct relationship between authentic leadership and turnover intention (Azanza et al., 2015). Although scholars have shown evidence of a significant relationship between authentic leadership and employee turnover intention, recent debates on authentic leadership suggested that more research in diverse contexts is needed (Gardner et al., 2021). For example, research contexts have been limited to only specific workforces, such as those in nursing and the hospitality industry (Oh and Oh, 2017). In addition, a large number of studies have been conducted mostly in the Western context, examining the effects of authentic leadership on several outcomes (see Gardner et al., 2011; Lemoine et al., 2019 for reviews). Therefore, further work examining the effects of authentic leadership on turnover intention in different contexts (e.g., non-Western context and non-hospitality industry) is necessary to assess the generalizability of the results. Turnover scholars also have argued that the field of turnover research would benefit greatly by looking at the generalizability of the existing turnover models to non-Western cultures (Holtom et al., 2008). In the present study, therefore, we examine the processes by which authentic leadership influences employee turnover intention (Yammarino et al., 2008). Moreover, given that the turnover process is complicated (Holtom et al., 2008), we propose that the relationship between authentic leadership and employee turnover intention might be influenced by certain situational factors such as organizational identification.

Authentic leadership theory rests on the assumption that this leadership style can inspire followers to enact a higher level of positive behaviors (Avolio et al., 2004). However, it is surprising that so little is known about the processes by which authentic leaders influence organizational level constructs such as perceived organizational support, organizational identification, and more importantly employee turnover intention. Scholars called for better understanding of the underlying mechanism by which authentic leadership affects organizational level outcomes (Yammarino et al., 2008). Subsequently, there have been some studies to examine the relationship between authentic leadership and organizational outcomes. For example, scholars found that authentic leadership is positively associated with organizational level constructs such as employees’ affective attachment to and organizational identification (Leroy et al., 2012). Most importantly, studies revealed that organizational identification mediates the relationship between authentic leadership and employee resilience using a theoretical framework that organizational identification can be influenced by their leaders (e.g., Mao et al., 2022). In this study, we aim to gain a better understanding of how authentic leaders reduce employee turnover intention. First, we argue that perceived supervisor support (PSS) is an important mechanism by which authentic leadership affects employee turnover intention. Organizational support theory suggests that the extent to which an organization or supervisor appears to care about an employee’s well-being influences the employees’ opinions about working for the organization (Kottke and Sharafinski, 1988). Furthermore, these opinions have positive relationships with employee commitment and performance (Rhoades and Eisenberger, 2002). The present study argues that by acting upon high-level authentic values and beliefs, authentic leaders can make followers believe that leaders support them through acts of authenticity, transparency, and balanced processing. With this theoretical framework, we suggest that supervisors’ authentic leadership is positively associated with PSS and thus leads to lower employee turnover intention. To the best of our knowledge, no research has explored the relationships between supervisors’ authentic leadership and PSS. By focusing on PSS as an intervening mechanism linking supervisors’ authentic leadership to employee turnover intention, we hope to contribute to the authentic leadership literature.

Second, we argue that the mediating effect of PSS in the relationship between authentic leadership and employee turnover intention is moderated by organizational identification. Transformational leadership and other leadership behaviors have been argued to be particularly effective at reducing employee turnover intention (Wang et al., 2011; Lemoine et al., 2019; Bedi, 2020). However, the empirical evidence of the role of authentic leadership in reducing employee turnover intention is inconsistent. For example, scholars have found a negative relationship between authentic leadership and employee turnover intention (Laschinger et al., 2012; Laschinger and Fida, 2014; Azanza et al., 2015; Olaniyan and Hystad, 2016). However, scholars have also shown that authentic leadership is positively directly related to intent to leave (Shapira-Lishchinsky and Tsemach, 2014). One explanation for the mixed results could be the presence of moderator variables. Indeed, too little is known about the impact of situational factors on the effects of authentic leadership on employee turnover intention. In this study, we suggest that organizational identification might be one possible moderator in this relationship. Organizational identification has been reported to be negatively related to turnover and turnover intention (Mael and Ashforth, 1995; Van Knippenberg and Van Schie, 2000; Van Dick et al., 2004; Cole and Bruch, 2006). Social identity theory (Ashforth and Mael, 1989) posits that organizational identification is the feeling of psychological oneness with the organization. Thus, regarding the role of organizational identification in the turnover process, people feel a strong sense of “sharing a common fate” with the organization whenever they identify strongly with that organization (Van Dick et al., 2004) and these feelings prevent individuals from quitting the organization or from having the intention to quit.

**Figure 1:**
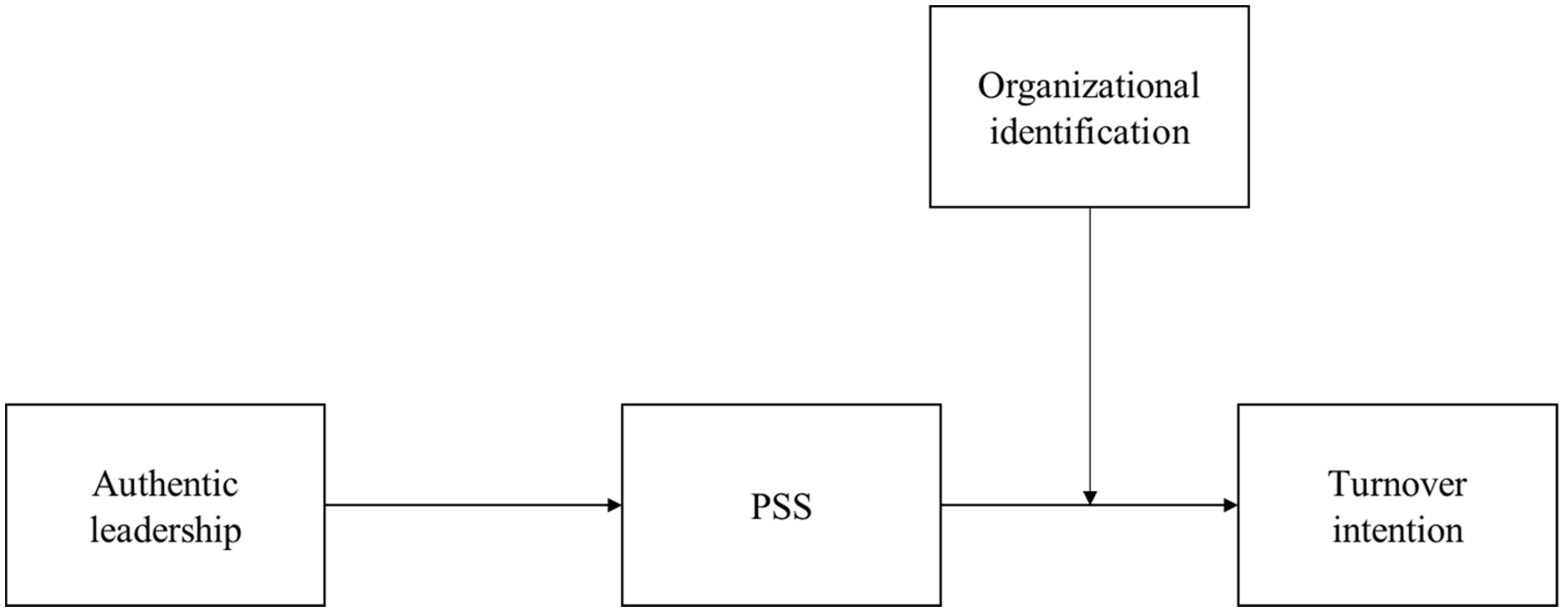
Proposed research model.

The purpose of our study is to examine how authentic leadership affects employee turnover intention using 433 employees from organizations in South Korea. First, drawing on organizational support theory and justice literature, we propose that PSS is positively associated with authentic leadership. Furthermore, we introduce PSS as an intermediate mechanism linking authentic leadership to employee turnover intention. Second, drawing on social identity theory, we examine how the relationship between authentic leadership and employee turnover intention is affected by a certain situational factor. Specifically, we argue that organizational identification might influence the relationship between PSS and employee turnover intention. In short, according to our model (see Figure 1), authentic leadership increases employees’ PSS level, which in turn reduces their turnover intention. Moreover, organizational identification moderates the relationship between PSS and turnover intention. Our study contributes to literature on authentic leadership and turnover intention in three ways. First, our study contributes to existing literature by assessing the generalizability of previous research outcomes on the relationship between authentic leadership and turnover using data from several different workforces in different cultures. Specifically, our study tests hypotheses using data from employees of organizations in South Korea because key aspects of authentic leadership might be more suitable for the East Asian context (Joo et al., 2016). Second, drawing on organizational support theory, our study also contributes to organizational support literature by introducing PSS as an intermediate mechanism in the turnover process by which authentic leadership influences employee turnover. Specifically, our study suggests that authentic leadership is an effective leadership style because it causes employees to believe that their supervisors support them significantly. Third, consistent with prior research on organizational identification (Abbasi et al., 2021; Ciampa et al., 2021; De Clercq and Belausteguigoitia, 2022), this study contributes to organizational identification literature by suggesting that identification might be a critical situational factor in the relationship between PSS and employee turnover intention.

## Theoretical background and hypotheses development

### Authentic leadership and turnover intention

According to authentic leadership theory, authentic leadership is posited to build trust between leaders and followers (Gardner et al., 2005) and support followers’ positive self-development (Luthans and Avolio, 2003). Therefore, it is logical to suppose that authentic leadership is negatively related to turnover intention among employees. Avolio et al. (2004) theoretically suggested that authentic leadership can influence employees’ attitudes and behaviors. They proposed that an authentic leader can induce positive attitudes such as organizational commitment and involvement in employees by instilling several psychological constructs such as trust, positive emotions, and optimism. They also proposed that authentic leaders foster employee retention (Avolio and Gardner, 2005; Wong and Cummings, 2009). In this study, we also expect that authentic leadership is negatively associated with employee turnover intention because of the relational nature of authentic leadership. Scholars suggest that relational transparency of authentic leadership refers to the open and transparent way whereby authentic leaders share information, and ultimately build collaborative relationships with followers (Walumbwa et al., 2008). When leaders build the relationship followers in authentic way, their relationships tend to be high quality social exchange relationship which is characterized by high levels of respect, positive emotion, and trust (Ilies et al., 2005). Therefore, we hypothesize that balanced processing of information, relational transparency, values and words, and consistency in actions of authentic leaders might play a significant role to encourage employees to stay in the organization. Considering the role of leaders in the workplace who continuously interact with followers and thus significantly influence their attitudes and behaviors, we argue that authentic leaders build trust in leadership, and which in turn decreases employee turnover intention.

Recently, empirical studies have provided evidence that authentic leadership is significantly related to employee intention to stay at organizations. For example, scholars have shown that authentic leadership is negatively associated with employee intentions to leave organizations (Olaniyan and Hystad, 2016). With an emphasis on intermediate mechanisms, other studies have demonstrated that authentic leadership contributes to the retention of employees by reducing employees’ emotional exhaustion (Laschinger and Fida, 2014), increasing their levels of job satisfaction (Laschinger et al., 2012), increasing their levels of work engagement (Azanza et al., 2015), increasing their levels of affective commitment (Ausar et al., 2016), and creating a work environment based on fairness (Kiersch and Byrne, 2015). In keeping with these efforts, we also hypothesize that perceptions of authentic leadership negatively affect employees’ turnover intention.

*Hypothesis 1*: Perceptions of authentic leadership are negatively associated with employee turnover intention.

### Authentic leadership and PSS

In this study, we propose that perceptions of authentic leadership are positively associated with employees’ PSS. According to organizational support theory, PSS refers to the extent to which employees believe that their supervisors value their contributions and care about their well-being (Kottke and Sharafinski, 1988). The present study argues that by acting upon high-level authentic values and beliefs, authentic leaders can make employees believe that leaders support them through acts of authenticity, transparency, and balanced processing. Our theoretical framework for authentic leadership as an antecedent of PSS relies on two arguments: (1) the common theoretical foundations of authentic leadership and organizational justice in predicting employees’ PSS (suggesting authentic leadership as a fair leadership) and (2) the ability of authentic leadership behaviors to develop high-quality relationship with employees to influence their PSS (suggesting authentic leader-follower relationship as a high-quality leader-member relationship). In short, we argue that authentic leaders enhance employees’ justice perceptions, which are strong antecedents of PSS (Rhoades and Eisenberger, 2002) and they also gain the respect and trust of employees by building high-quality leader–follower relationships, which contribute to the development of employees’ PSS (Kurtessis et al., 2017).

First, authentic leadership is considered a fair leadership. Fairness theory (Folger et al., 2005) proposes that moral accountability is one of key features to the formation of justice judgments and perceptions. Similarly, morality is one of the central factors of being an authentic leader (e.g., May et al., 2003), and authentic leaders show integrity and moral virtue to followers at work (e.g., George, 2003; Luthans and Avolio, 2003). In sum, morality is one of central features of authentic leaders and is also a critical component of organizational justice. Thus, we propose that authentic leadership is a fair leadership because it should create perceptions of organizational fairness or justice. For example, procedural justice criteria (Leventhal, 1980) are met by authentic leaders because they are open and transparent when they make decisions (e.g., Avolio et al., 2009). Hence, employees experience these authentic leadership behaviors and then form positive fairness or justice perceptions accordingly, thus perceiving authentic leaders a kind of fair leaders. Specifically, we argue that authentic leaders improve employees’ interpersonal justice perceptions by building trusting relationships and gaining their respect, which are significant components of interpersonal justice, and they improve employees’ informational justice perceptions by sharing and analyzing all relevant information with them before making decisions, thereby showing their authenticity (Walumbwa et al., 2008; Gardner et al., 2011; Lemoine et al., 2019).

First, an authentic leader can show their authenticity and thereby improve the interpersonal justice perceptions of employees in the workplace by building trusting relationships with followers. As Kernis (2003) demonstrated, when individuals come to know themselves, they are more comfortable forming transparent, open, and close relationships with others. According to authentic leadership scholars, authentic leaders show enduring authenticity in their relationships with others. Thus, they are more likely to form transparent and open relationships with employees. In other words, this component of authentic leadership behavior involves pursuing openness and trust in relationships with followers (Ilies et al., 2005). For example, Avolio et al. (2004) theoretically suggest that authentic leaders gain credibility and followers’ trust by building collaborative relationships with followers. Furthermore, authentic leaders can improve informational justice perceptions by sharing information with employees. For example, Walumbwa et al. (2008) showed that authentic leaders can increase justice perceptions by enacting relationally transparent behaviors, such as openly sharing information with followers before coming to a decision (balanced processing), and fairness with regards to information processing. We conclude that authentic leaders increase employee justice perceptions by building relationships based on trust with employees and showing transparency during interactions with employees.

Empirical support has demonstrated the relationships between different types of justice and a broad range of employee outcomes (see Colquitt, 2008; Colquitt et al., 2013; Colquitt and Zipay, 2015; Lind, 2019, for reviews). For example, scholars have suggested that organizational justice (especially procedural and interactional justice) leads to the formation of social exchange relationships that involve socioemotional resources. Procedural justice also strengthens the emotional bond with the organization (Tyler and Lind, 1992). Chebat and Slusarczyk (2005) reported that fair interactions with supervisors were related to employees’ positive emotion. In addition, fair treatment by leaders (interactional justice) is also positively related to group members’ respect and pride (Lind and Tyler, 1988). Lastly, Judge et al. (2006) also found interpersonal justice to be negatively associated with hostility.

Among these outcomes, Rhoades and Eisenberger (2002) found that fairness of treatment was strongly related to employee’s perceived support in organizations. For example, repeated demonstrations of justice contribute to perceived support by showing concern for employees’ welfare (Shore and Shore, 1995). Fasolo (1995) reported that justice was positively related to PSS. Therefore, we predict that authentic leadership improves the justice perception of employees, and in turn, increases the level of PSS.

Second, besides the effect on justice perception, authentic leaders can develop high-quality leader–follower relationships. Specifically, we argue that the relationship between authentic leaders and followers is considered a type of high-quality LMX relationship. Avolio et al. (2004) advised us to “notice, however, that such an intimate, trusting and cooperative relationship is not possible without authenticity and the self-awareness, self-acceptance, and transparent conveyance of one’s actual, ought and ideal selves that accompany it” (p. 811). Therefore, we contend that some characteristics of an authentic leader, such as authenticity and transparency, help to establish a high-quality collaborative exchange relationship between leaders and followers. Furthermore, we assume that the perception of authentic leadership by followers contributes to their high LMX.

Social Exchange Theory has been used as the conceptual foundation for a large body of LMX research. Blau (1964) proposed that social exchanges are based on the belief that goodwill gestures will be reciprocated quickly. In accordance with Wayne et al. (1997), we argue that the perception of authentic leadership is a predictor of perceived support because employees tend to develop a high-quality exchange relationship with their supervisor if they perceive their supervisor as being an authentic leader. Eisenberger et al. (1986, 2002) theorized and meta-analytically proved that discretionary rewards by leaders affect perceived support. Because leaders are often charged with the distribution of such rewards, we expect that LMX may contribute to PSS (Settoon et al., 1996; Wayne et al., 1997; Hofmann and Morgeson, 1999). Therefore, we believe that authentic leadership is positively associated with PSS.

Taken together, we hypothesize that authentic leadership is positively associated with PSS because authentic leadership is considered fair leadership by followers and they are likely to develop a high-quality relationship with their supervisor if that supervisor is supportive and fair and is thus perceived as an authentic leader.

*Hypothesis 2*: Perceptions of authentic leadership are positively associated with employees’ perceived supervisor support.

### Mediating effect of PSS

Responding to a call for inquiry of possible mechanisms in the relationship between authentic leadership and employee outcomes (Avolio et al., 2004), scholars have investigated the processes by which authentic leadership affects employees’ work outcomes through their perceptions, cognition, attitudes, and behavior. For example, in a study that examined the effect of authentic leadership on followers’ proactive behavior, scholars found the significant mediating effect of follower psychological capital and the significant moderating effect of compassion at work of the relationship between authentic leadership and follower proactive behavior (Hu et al., 2018). Scholars have also found that organizational commitment mediates the relationship between authentic leadership and follower creativity (Ribeiro et al., 2020). Lastly, other scholars found that authentic leadership reduces leaders’ stress and increases their work engagement and that these effects are mediated by leader mental depletion (Weiss et al., 2018).

Given the importance of processes to fully understand how authentic leadership affects employees in the workplace, we argue that it is necessary to identify intermediate mechanisms by which authentic leaders influence employee turnover intention, as presented in Figure 1. We propose PSS as an intervening variable to explain how authentic leadership enhances the overall perception concerning the extent to which the supervisor values the contributions of employees and cares about their well-being. These perceptions, rooted in the principles of social exchange (Blau, 1964), ultimately negatively influence employees’ intention to leave their organization. Social Exchange Theory views employment as a social exchange relationship (Blau, 1964). The employee trades effort and loyalty for tangible benefits and social resources from the organization (Cropanzano and Mitchell, 2005). Unlike an economic relationship where physical goods are exchanged, this social exchange relationship is characterized by mutual reciprocity (Gouldner, 1960) of social and emotional benefits (Van Dyne et al., 1994). Comparing the employment relationship with the social exchange, where personal efforts and loyalty are rewarded physically and socially, we can see the organizational commitment from the social exchange perspective, that is, organizational members express commitment in return for support from the organization (Eisenberger et al., 1986; Rhoades et al., 2001). Besides commitment, with the norm of reciprocity, many study results have shown that there are significant relationships between POS and important outcomes at the individual and organizational level (e.g., Settoon et al., 1996; Wayne et al., 1997; Eisenberger et al., 2001, 2002; Rhoades et al., 2001; Rhoades and Eisenberger, 2002).

Besides POS, PSS has been found to be associated with important individual and organizational outcomes, such as employee retention (Eisenberger et al., 2002), in-role and extra-role behaviors (Shanock and Eisenberger, 2006; Gkorezis, 2015; Potipiroon and Ford, 2019), and turnover (Maertz et al., 2007). Because supervisors are viewed as representatives of the organization, employees view their supervisor’s favorable or unfavorable attitudes toward them as an indicator of the organization’s level of support for them (Eisenberger et al., 1986). Therefore, similar to most research on POS, research on PSS has mainly invoked social exchange theory to explain the reciprocity relationship resulting from a high level of PSS. In addition, PSS has also been examined as a job resource since supervisors have the power to allocate job resources to employees (Jokisaari and Nurmi, 2009). According to Conservation of Resources (COR) theory (Hobfoll, 1989), when employees perceive support from the supervisor, employees will be more likely to access opportunities and resources in the workplace. In various studies, supervisor support has been found to be positively related to intent to stay (Sourdif, 2004; Lu et al., 2005; Lynn and Redman, 2005; Nedd, 2006; Tourangeau and Cranley, 2006; Lacey et al., 2007; Chen et al., 2008).

Concerning voluntary employee turnover, although some researchers have argued that when PSS is low, employees deal with the problem by switching to another supervisor in the same organization (Kurtessis et al., 2017), a meta-analysis on PSS shows a negative relationship between PSS and turnover intentions (*r* = −0.29, *p* < 0.001), especially for boundary-spanning employees (Edmondson and Boyer, 2013). From the social exchange perspective, PSS involves employees into the reciprocal relationship which leads to their lower intention to leave. From the job resource perspective, as supervisors play the key role in providing job resources, employees are more likely to stay in an organization when the benefits received from relationships with supervisors outweigh the costs (Cook and Emerson, 1978). Especially, benefits obtained from the relationship are increased when leaders show authenticity, which enhances employees’ PSS. Therefore, authentic leadership will negatively impact an employee’s turnover intention by increasing employees’ PSS. Taken together, we develop the following hypothesis:

*Hypothesis 3*: Employees’ perceived supervisor support mediates the relationship between perceptions of authentic leadership and employee turnover intention.

### Moderating effects of organizational identification

Scholars have given increasing consideration of moderators in the employee turnover process because the relationship between turnover and its antecedents and outcomes is not clear, indicating that the turnover process is complex (Holtom et al., 2008). For example, the performance–turnover relationship is moderated by salary growth and promotions and is more pronounced for employees with lower salary growth and employees given promotions (Trevor et al., 1997). Trevor (2001) also found that general job availability, movement capital, and job satisfaction interact with each other simultaneously to affect turnover. The relationship between turnover intentions and turnover is moderated by various personality traits, such that the impact of the relationship is stronger for employees with low self-monitoring, low risk aversion, and an internal locus of control (Allen et al., 2005).

Because the turnover process is complex, in this study, we argue that the effects of authentic leadership on turnover intention are strengthened or weakened by certain factors. Scholars have already found that the negative indirect effects of authentic leadership on employees’ turnover intentions by changing the levels of affective commitment are significant (Oh and Oh, 2017). Furthermore, they found that organizational size moderates the mediated relationship between authentic leadership and turnover intentions, such that the relationship is stronger in smaller organizations.

In this study, we argue that when employees feel strong support from their supervisor (i.e., PSS), it is highly likely that they will form emotional attachments and commit more strongly to their own group or organization, leading to lower turnover intention. However, certain variables can impact the negative relationship between PSS and turnover intention. Although some scholars suggested organizational identification as an intervening variable of the relationship between authentic leadership and outcomes (Mao et al., 2022), we propose that organizational identification plays an important moderating role in the effect of authentic leadership on turnover intention. Social identity theory suggests that organizational identification can contribute to employees’ attitudes and behaviors such as OCB, job satisfaction, commitment, and turnover intention. Therefore, the relationship between PSS and turnover intention is likely to be strengthened to the extent that employees identify themselves with their organizations.

Organizational identification is “a sense of linkage, and a psychological state wherein an individual perceives himself or herself to be part of a larger whole (work group, firm, church, etc.)” (Rousseau, 1998, p. 217). Social identity theory proposes that an individual’s identity is determined by their individual traits and by the knowledge of their membership in a group or organization (Tajfel and Turner, 1979). Therefore, favoritism toward in-group members and discrimination against non-members naturally occur.

To date, various studies have explored the changes in attitudes or behaviors brought by organizational identification. At the individual level, the outcomes of organizational identification include organization-based self-esteem (Bergami and Bagozzi, 2000), positive attitudes toward the organization (Ashforth and Mael, 1989), reduced uncertainty (Hogg, 2000), and self-verification (Polzer et al., 2002). Organizational identification is associated with affiliation (Dutton et al., 1994), job satisfaction, organizational commitment, and subordinates’ other positive attitudes (Butler et al., 1999; Dirks and Ferrin, 2002). Organizational identification may induce some specific behaviors in individuals and become a basis for certain behaviors at both the individual and the organizational level (Lee et al., 2015; Blader et al., 2017). Many studies have reported that organizational identification is related to some specific behaviors, such as cooperative behavior and OCB (Dukerich et al., 2002; Van Dick et al., 2006).

In this study, the relationship between PSS and turnover intention is hypothesized to depend on the strength of organizational identification because organizational identification significantly affects employee turnover intention by improving employee job satisfaction (e.g., Van Dick et al., 2004; Carmeli et al., 2007). Social identity theory (Ashforth and Mael, 1989) suggests that organizational identification is the feeling of cognitive or psychological oneness with an organization. Therefore, organizational members who show strong identification with an organization are likely to have feelings of “common fate” with that organization (Van Dick et al., 2004). These feelings deter individuals from quitting the organization or from having the intention to quit. Thus, we believe that organizational identification is positively related to job satisfaction and in turn makes employees more likely to remain in organizations. Specifically, scholars have suggested that people who strongly identify with their organizations also feel more positive toward their job, leading to higher job satisfaction due to the heightened self-esteem instilled *via* strong identification (Bergami and Bagozzi, 2000; Hogg and Terry, 2001; Judge and Bono, 2001). Empirical studies have also reported that organizational identification has negative associations with turnover and turnover intention (Van Knippenberg and Van Schie, 2000; Van Dick et al., 2004; Cole and Bruch, 2006) when subordinates identify themselves with their organizations, they will be more likely to stay.

Taken together, we hypothesize that organizational identification strengthens the relationship between PSS and turnover intention, and that the strength of organizational identification significantly affects this relationship:

*Hypothesis 4*: Organizational identification moderates the relationship between employees’ perceived supervisor support and employee turnover intention, such that a negative association between perceived supervisor support and employee turnover intention is stronger for employees with a higher level of organizational identification.

### Moderated mediation

Hypothesis 2 states that PSS has a mediating role in the relationship between authentic leadership and turnover intention. In addition, Hypothesis 3 illustrates the moderating effect of organizational identification on the authentic leadership–turnover intention relationship. Hypotheses 1–3 suggest that authentic leadership affects employee turnover intention *via* PSS, with this mediation effect being moderated by organizational identification, which can be represented by a moderated mediation model (Edwards and Lambert, 2007). Therefore, by combining these hypotheses, we propose that organizational identification intensifies the mediating effect of PSS on the relationship between authentic leadership and turnover intention. Thus, employees who strongly identify themselves with their organization are more likely to feel common fate with organizations and thus remain with their organization. Accordingly, the indirect effect of authentic leadership on turnover intention should be stronger. Thus, we propose the following hypothesis.

*Hypothesis 5*: Organizational identification moderates the mediating effect of perceived supervisor support on the relationships between perceptions of authentic leadership and employee turnover intention, such that the mediating effect is stronger when the level of organizational identification is high.

## Materials and methods

### Participants and procedure

We adopted convenience sampling to recruit survey respondents working in several organizations because of its convenience and accessibility. Specifically, we approached 500 South Korean employees working on a full-time basis at several companies, for example, a bank, an airline company, and a research institute. We informed that participation was voluntary, and respondents could refuse to participate in this study without any consequence. For example, we attached a cover letter to the survey to inform participants that participation was voluntary, that the survey was anonymous, that any data would only be used for research purposes, and that their responses would be confidential. In addition, we notified that participants could withdraw at any time during the survey process. We explained the scope of the research to all participants to ensure that their decision to participate was voluntary. After obtaining consent from employees in each organization, we distributed questionnaires to employees.

We used a self-report research design to investigate the effect of supervisor’s authentic leadership on employee turnover intention. Specifically, the survey was administered with self-reports mainly based on respondents’ perceptions about key variables including supervisors’ authentic leadership, their PSS level, organizational identification and turnover intention. In order to reduce the common method variance problem in self-report research design, we conducted a two-phase survey in which two waves of data collection were separated by approximately 3 weeks (Donaldson and Grant-Vallone, 2002). In the first phase, a total of 500 hard copy questionnaires were distributed to participants in several organizations. They were asked to answer questions about their supervisors’ authentic leadership, their level of PSS, and other demographic information (Time 1). Four hundred eighty usable survey questionnaires were collected with a response rate of 83.33%. In the second phase, 480 hard copy of questionnaires were delivered to respondents who already completed their survey questionnaire in the first phase. They were asked to rate the level of turnover intention over the past weeks (Time 2). A total of 450 questionnaires were returned, corresponding to a response rate of 93.8%. After removing the inattentive responses, we retained 433 valid and completed questionnaires for data analysis.

Of 433 respondents, 57% of the participants were male and 87% of the participants had experience working full-time. Considering job roles, the participants comprised ordinary employees (35%), deputy section chiefs (27%), section heads (22%), and department heads or board members (16%). The tenures of participants at their respective organizations were as follows: more than 7 years (32%), more than 5 years but less than 7 years (18%), more than 3 years but less than 5 years (25%), and less than 3 years (24%). The industries included the service industry (31%), manufacturing (22%), and the financial industry (21%). In terms of the number of employees at organizations, 64% of the organizations had more than 2,000 employees, and 27% had less than 500 employees.

### Measures

The original English survey items were translated into Korean by two Korean bilingual researchers of our research team independently. The translation-back-translation method (Brislin, 1980) was used to ensure the accuracy of the translation. We did the pilot test before formally distributing the questionnaire to ensure its clarity and robustness of items. The pretest on 10 employees from the participating organization did not reveal major problems in understanding the questionnaire items. Unless indicated, measures presented in this study were based on a 5-point Likert scale ranging from 1, “strongly disagree,” to 5, “strongly agree.”

#### Authentic leadership

Supervisor’s authentic leadership was measured by 16 items developed by Walumbwa et al. (2008). Respondents were asked to rate the authentic leadership of their immediate supervisor using 16 items. A sample item is as follows: “My leader says exactly what he/she means.” The Cronbach’s alpha for authentic leadership for this study was 0.87.

#### Perceived supervisor support

PSS was measured by eight items developed by Eisenberger et al. (2002). This shorter version of PSS was used in this study because many scholars have used this version without any reliability problems (Rhoades and Eisenberger, 2002). A sample item is as follows: “The supervisor really cares about my well-being.” The Cronbach’s alpha for PSS for this study was 0.90.

#### Organizational identification

We assessed organizational identification with six items developed by Mael and Ashforth (1992). A sample item is as follows: “When someone praises this company, it feels like a personal compliment.” The Cronbach’s alpha for organizational identification for this study was 0.88.

#### Turnover intention

We measured turnover intention by using three items developed by Konovsky and Cropanzano (1991). A sample item is as follows: “I intend to look for a job outside of my organization within the next year.” The Cronbach’s alpha for turnover intention for this study was 0.80.

#### Control variables

We controlled for variables that were previously shown to be associated with variables in preceding studies (Holtom et al., 2008; Hom et al., 2012). Those were gender, rank, status, tenure, education, firm size, job satisfaction, and job stress. Gender was coded 1 for “male” and 2 for “female.” Rank was coded 1 for “ordinary employees,” 2 for “deputy section chiefs,” 3 for “section heads” and 4 for “department heads or board members.” Employment status was coded 1 for “full-time” and 2 for “part-time.” Tenure was coded 1 for “less than 3 years,” 2 for “more than 3 years but less than 5 years,” 3 for “more than 5 years but less than 7 years,” and 4 for “more than 7 years.” Firm size was coded based on the number of employees at organizations, 1 for “less than 500 employees,” 2 for “500 to 1,000 employees,” 3 for “1,000 to 2,000 employees”, and 4 for “above 2,000 employees.” Job satisfaction was measured with 3-item scale developed by Bono and Judge (2003) and job stress was measured with a 4-item scale developed by Keller (2001). Responses range from 1, “strongly disagree” to 5, “strongly agree.”

## Results

### Preliminary analysis

We conducted a series of confirmatory factor analyzes (CFA) to test the factor structure of the study variables and to prepare the data for subsequent correlation and regression analyzes using AMOS 21 (see Table 1). First, we performed a CFA for four constructs (i.e., authentic leadership, PSS, organizational identification, turnover intention). This allowed to determine the fit of the hypothesized four-factor models before testing the hypotheses. As shown in Table 1, the fit indices suggested a good fit of the model (*χ*^2^/df = 1.94, RMSEA = 0.05, NFI = 0.909, IFI = 0.954, TLI = 0.947 and CFI = 0.953). Factor loadings of less than 0.5 (Hair et al., 2010) were deleted (AL5; PSS6). The remaining item loadings were higher than the recommended threshold, suggesting acceptable convergent validity. Second, a three-factor model was tested by combining PSS and organizational identification (*χ*^2^/df = 4.86, RMSEA = 0.10, NFI = 0.761, IFI = 0.800, TLI = 0.783, and CFI = 0.799). Finally, a two-factor model was examined by loading all survey items other than authentic leadership on one factor (*χ*^2^/df = 5.44, RMSEA = 0.10, NFI = 0.731, IFI = 0.769, TLI = 0.751 and CFI = 0.768). Overall, the fit indices suggested a better fit of hypothesized four-factor model than other models.

**Table 1:** Confirmatory factor analyzes of the measurement model.

Models	Factors	*χ* ^2^	DF	*χ*^2^/DF	RMSEA	NFI	IFI	TLI	CFI
Hypothesized model	Four-factor model	797.89	412.00	1.937	0.046	0.909	0.954	0.947	0.953
	Three-factor model	2094.15	431.00	4.859	0.095	0.761	0.800	0.783	0.799
	Two-factor model	2354.99	433.00	5.439	0.101	0.731	0.769	0.751	0.768

*N* = 433.

### Tests of hypothesis

Table 2 presents the means and standard deviations for all variables, as well as the inter-correlations between them. To presentational parsimony, we present results of regression without controls but provide their bivariate correlations with variables for this study in Table 2. Authentic leadership is negatively and significantly correlated with turnover intention (*r* = −0.37, *p* < 0.01), and positively and significantly correlated with PSS (*r* = 0.74, *p* < 0.01). PSS is also negatively and significantly correlated with turnover intention (*r* = −0.47, *p* < 0.01).

**Table 2:** Correlations and descriptive statistics.

Variables	*M*	*SD*	1	2	3	4	5	6	7	8	9	10	11
1. Gender	1.43	0.5											
2. Status	1.12	0.33	0.31^**^										
3. Rank	2.32	1.34	−0.29^**^	−0.34^**^									
4. Tenure	2.59	1.17	−0.18^**^	−0.39^**^	0.62^**^								
5. Firms size	3.07	1.33	−0.20^**^	−0.06	−0.12^*^	0.05							
6. Job satisfaction	3.58	0.71	−0.11^*^	−0.1	0.27^**^	0.17^**^	−0.18^**^						
7. Job stress	3.56	0.7	−0.01	0.04	−0.05	−0.09	0.14^**^	−0.27^**^					
8. Authentic leadership	3.24	0.75	−0.12^*^	−0.08	0.06	0.07	−0.05	0.47^**^	−0.04				
9. PSS	3.24	0.73	−0.10^*^	−0.13^*^	0.14^**^	0.15^**^	−0.13^**^	0.62^**^	−0.10^*^	0.74^**^			
10. Organizational identification	3.54	0.76	−0.11^*^	−0.07	0.18^**^	0.17^**^	−0.04	0.49^**^	−0.06	0.34^**^	0.41^**^		
11. Turnover intention	2.78	0.93	0.12^*^	0.12^*^	−0.16^**^	−0.15^**^	0.03	−0.57^**^	0.28^**^	−0.37^**^	−0.47^**^	−0.41^**^	

*N* = 433. *p < 0.05; **p < 0.01.

To test our hypotheses, we conducted a regression analysis. Tables 3, 4 summarize the results of the regression analysis. In Table 3, authentic leadership was found to be negatively related to turnover intention (*β* = −0.13, *p* < 0.01), and positively related to PSS (*β* = 0.59, *p* < 0.01), thus supporting Hypotheses 1 and 2.

**Table 3:** Regression analyzes for Hypotheses 1 and 2.

Predictor variables	DV = PSS			DV = Turnover Intention					
	Model 1			Model 2			Model 3		
	*B*	SE B	𝛽	*B*	SE B	𝛽	*B*	SE B	𝛽
Authentic leadership	0.59	0.03	0.59^**^	−0.17	0.06	−0.13^**^	−0.01	0.08	−0.01
PSS							−0.27	0.09	−0.22^**^
Adjusted *R*^2^		0.68			0.34			0.36	
∆*R*^2^		0.68^**^			0.36^**^			0.02^**^	

*N* = 433, ^*^p < 0.05, ^**^p < 0.01.

**Table 4:** Regression analyzes for Hypothesis 3.

Predictor variables	DV = Turnover Intention					
	Model 4			Model 5		
	*B*	SE B	𝛽	*B*	SE B	𝛽
Authentic leadership	−0.03	0.08	−0.02	−0.04	0.08	−0.03
PSS	−0.22	0.09	−0.18^*^	0.42	0.26	0.34
Organizational identification	−0.18	0.06	−0.15^**^	0.38	0.22	0.30
PSS × Organizational identification				−0.18	0.07	−0.81^**^
Adjusted *R*^2^		0.37			0.38	
∆*R*^2^		0.39^**^			0.01^*^	

*N* = 433, ^*^p < 0.05, ^**^p < 0.0.

To test mediation hypothesis, we followed procedures suggested by Kenny et al. (1998). Hypothesis 3 predicted that there will be mediating effects of PSS on the relationship between authentic leadership and turnover intention. Our analysis revealed that when PSS was included in the figures in Model 3 of Table 3, authentic leadership’s effects disappeared compared with Model 2 (*β* = −0.13, *p* < 0.01 → *β* = −0.0.01, n.s.). Thus, PSS completely mediates between authentic leadership and turnover intention, supporting Hypothesis 3.

We also conducted a bootstrapping-based mediation test using the PROCESS macro in SPSS 25 to verify Hypothesis 3. We estimated the indirect effect of authentic leadership on employee turnover intention *via* PSS using unstandardized coefficients and a bootstrapping procedure with 5,000 resamples to produce a 95% confidence interval around the estimated indirect effects (Preacher and Hayes, 2008). The bootstrapped indirect effect is significant if the bias-corrected 95% confidence interval (CI) excludes zero. Our analysis revealed that authentic leadership is associated with decreased turnover intention, mediated by PSS (*indirect effect* = −0.16, SE =0.07, 95%CI = −0.31 to −0.02; *direct effect* = 0.008, SE = 0.08, 95% CI = −0.17 to 0.15; *total effect* = −0.17, SE = 0.06, 95% CI = −0.28 to −0.05). Hence, Hypothesis 3 is verified with a bootstrapping-based mediation test too.

**Figure 2:**
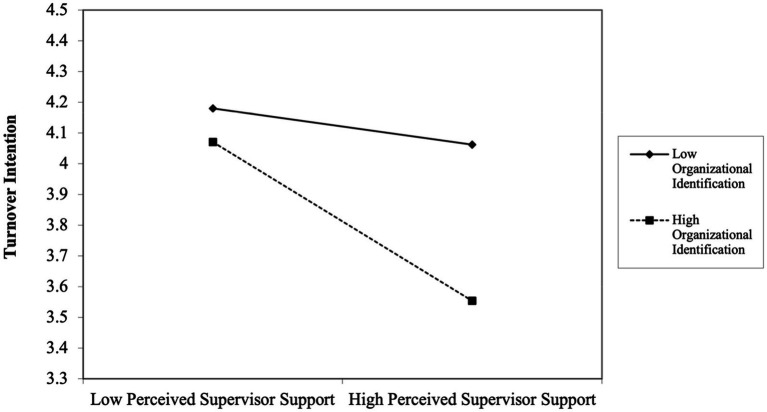
Interaction effect of organizational identification.

Hypothesis 4 states that organizational identification will moderate the relationship between PSS and employee turnover intention. Model 5 in Table 4 shows that the interaction between PSS and organization identification on turnover intention is negative and significant (*β* = −0.81, *p* < 0.01). To interpret this moderating effect, we re-arranged multiple regression equation into simple regressions, given conditional values of organizational identification (mean + 1 s.d.; mean – 1 s.d.; *cf.* Aiken and West, 1991). This relationship is plotted in Figure 2. In Figure 2, as predicted, the plots of the interaction terms show that PSS was related to turnover intention for individuals in high organizational identification. In contrast, the flat slope shows that PSS did not affect turnover intention for individuals in low organizational identification. Thus, this pattern of results lends support to Hypothesis 4.

Finally, we used Process macro in SPSS 25 to test Hypothesis 5 (Table 5). The results based on 5,000 resamples suggest that the indirect effect of authentic leadership on employee turnover intention is statistically significant at high levels of moderation (*conditional indirect effect* = −0.19, SE = 0.08, 95% CI = −0.334 to −0.033) but not at low levels (*conditional indirect effect* = −0.05, SE = 0.08, 95% CI = −0.211 to 0.110). We calculated the index of moderated mediation to assess the statistical significance of the moderated mediation effect. The index was significant (*Index* = −0.11, SE = 0.04, 95% CI = −0.187 to −0.019). These analyzes therefore suggest support for our overall moderated mediation hypothesis (Hypothesis 5).

**Table 5:** Summary of indirect effects and conditional indirect effects.

Paths and effects	Effect	SE	95% Confidence interval	
			Lower	Upper
**Authentic leadership → PSS → Turnover intention**				
Indirect effects	−0.16	0.07	−0.306	−0.015
Moderated mediation				
High organizational identification	−0.19	0.08	−0.334	−0.033
Low organizational identification	−0.05	0.08	−0.211	0.110
Indirect difference	−0.11	0.04	−0.187	−0.019

## Discussion

This study developed and tested a moderated mediation model that shows how, and under what conditions, authentic leadership is associated with employees’ turnover intention using data from participants in business organizations. Overall, our analysis shows that authentic leadership has a significant negative effect on employee turnover intention. Specifically, we found that authentic leadership is indirectly related to employees’ turnover intention through increased PSS, with significant mediating effects. Furthermore, we analyzed the moderating role of organizational identification and found that the negative relationship between PSS and turnover intention is stronger for individuals with high organizational identification.

### Implications for research

In line with previous studies on authentic leadership (Gardner et al., 2011; Lemoine et al., 2019), our study explained how authentic leadership influences employee turnover intention through a mediating mechanism. Although previous studies on authentic leadership have found important psychological mechanisms linking authentic leadership to individual and organizational outcomes, our study contributes to the authentic leadership literature by introducing PSS as a mediator in the effect of authentic leadership on employees’ turnover intention. In this study, we did additional empirical research to investigate more complicated mechanisms by which authentic leadership influences employee outcomes, as well as to expand authentic leadership theory’s nomological network by introducing organizational support theory (OST) to explain the mechanism by which the authentic leadership decreases turnover intention (Gardner et al., 2011).

This research has important theoretical implications. Our findings extend the previous research in three important ways. First, we found a negative relationship between authentic leadership and turnover intention using data collected from several different organizations in Korea. As Avolio and Gardner (2005) assert, authentic leadership is the root construct of positive forms of leadership. In addition, authentic leaders show enduring authenticity in their relationships with employees, and exhibit authentic behavior or actions (Walumbwa et al., 2008). Therefore, scholars have shown that authentic leadership is negatively associated with employee turnover by affecting employees positively (Ausar et al., 2016; Oh and Oh, 2017). However, as scholars argue, further work examining the effects of authentic leadership on turnover intention in different contexts is necessary to assess the generalizability of the results (Holtom et al., 2008; Oh and Oh, 2017). In this study, we confirm previous research findings using data from diverse workforces in several organizations of one Asia country. Thus, our study contributes to turnover research and authentic leadership research by looking at the generalizability of the existing literature on the relationship between authentic leadership and turnover.

Second, we found PSS to be an important mediating variable in the relationship between authentic leadership and turnover intention. To provide additional research to investigate the mechanisms between authentic leadership and outcomes (Avolio et al., 2004; Gardner et al., 2011), we examined the role of PSS in this relationship and found the significant mediating role of PSS in explaining how authentic leadership influences employees’ turnover intention. Our study showed that authentic leadership *via* a fair leader who can develop high-quality exchange relationships with employees could enhance employees’ perceived support from supervisors. Leadership scholars suggest that the effects of authentic leaders on employees might be more powerful through some mechanisms (Avolio et al., 2004). Therefore, previous studies have investigated several mechanisms by which authentic leadership affects employee outcomes. For example, trust in leader is suggested one of positive factors in the relationship between authentic leadership and employee outcomes (Maximo et al., 2019; Qiu et al., 2019). Scholars also found that authentic leadership indirectly affects team performance (Lyubovnikova et al., 2017). In short, given the importance of organizational and supervisory support in the relationship between leadership and employee outcomes (see Kurtessis et al., 2017; Thompson et al., 2020 for reviews), our study contributes to both the Organizational Support Theory (OST) and leadership literature by adding PSS as one of the mediating mechanisms in the relationship between authentic leadership and turnover intention.

Lastly, the second objective of this research was to investigate whether employee organizational identification acts as a situational factor affecting the relationship between authentic leadership and employee turnover intention. Consistent with prior studies on moderating roles of organizational identification in the workplace (De Cremer, 2005; Lipponen et al., 2008; Tangirala and Ramanujam, 2008; Abbasi et al., 2021; Ciampa et al., 2021; De Clercq and Belausteguigoitia, 2022), our research highlights the role of organizational identification in facilitating PSS into employee turnover intention. Specifically, we found organizational identification to be a situational factor that might influence the relationship between authentic leadership and turnover intention. To the best of our knowledge, no other study has investigated how organizational identification contributes to the relationship between authentic leadership and turnover intention. Thus, we add to the existing identification and leadership literature by showing that organizational identification strengthens the negative effect of authentic leadership on turnover intention.

### Implications for practice

Our empirical findings have many practical implications for leaders and organizations. First, turnover intention has drawn so much attention mainly because of the direct high cost of voluntary turnover behavior as well as indirect costs due to its effect on organizations (Hogan, 1992). According to our research results, leaders who are authentic to themselves, show enduring authenticity in relationships with employees, and enact authentic behaviors or actions are especially necessary enhance employee’s perceived support and mitigate turnover intention, which will finally contribute to organization performance. Second, recent studies found that employees working during the pandemic were highly impacted due to various and unusual workplace circumstances (Bufquin et al., 2021; Huffman et al., 2022). Social support has been shown to be a buffer against the effects of the pandemic among different age groups (Li et al., 2021). Our findings carry important implications for managers and organizations looking for a resilient pandemic recovery. For managers, knowledge of how to keep valued employees and how to motivate them might become key issues in the post-pandemic future. According to our research results, authentic leadership are beneficial to enhance employee perceived support and alleviate negative feelings and actions among employees in today’s turbulent business environment. Third, another shift in business history induces a public desire for authentic leadership besides the economic crisis (Goffee and Jones, 2005). Technological advancement allows people to access historical and real-time information online easily. As a result, the public can assess the authenticity of a leader by checking their past information. Therefore, fraudulent scandals witnessed by the public can be attributed to unauthentic leaders who previously failed to fulfill their obligations. As such, business leaders face being scrutinized by the public and the confidence in them decreasing (Case et al., 2018). Corporate social responsibility, as a result, is becoming more important. “People want to be led by someone ‘real.’” (Goffee and Jones, 2005, p. 86) The more transparency the public desires, the more aware leaders become regarding their authenticity and the more authentic leadership is expected. To gain the trust of employees as well as the public, leaders’ actions need to be authentic, and leaders must consider the long-term effects of their actions.

Fourth, our results highlight the positive effects of authentic leadership and show that authentic leaders are able to influence employees’ attitudes and behavior by increasing their employees’ PSS. Therefore, investing in leadership development programs to build a team of authentic leaders is advisable for organizations. According to the developmental perspective and theoretical foundation of authentic leadership (Luthans and Avolio, 2003), gifted with innate capabilities, leader’s perspectives, values and behaviors could also be established by positive organizational context and planned trigger events. We recommend that organizations should adopt a proactive, interventionist strategy in their leadership development program. With control over trigger events and their timing, leadership development program will contribute to the development of a sense of self-awareness and in turn, eventually build authentic leaders.

Moreover, because authentic leadership focuses on relationships with employees and the relational characteristics of authentic leaders can encourage employees to have positive feelings and attitudes and conduct positive behaviors, leadership development tools that focus on developing authentic characteristics, such as relational transparency and balanced processing, should be developed. Future research should continue to explore the effect of authentic leadership training and development practices on organizations in business environment.

Lastly, our study shows that organizational identification significantly strengthens the effect of PSS on negative outcomes. If employees do not strongly identify themselves with their organization, PSS is less influential. As the workforce has been more diversified in recent years, managers need to adapt traditional ways to motivate and retain employees. Therefore, we recommend that rather than retaining employees *via* extrinsic rewards, building an inclusive culture, and enhancing employees’ identification with the organization is preferable to better motivate them to remain at the organization.

## Conclusion

Using data from 433 employees from multiple industries in South Korea, our work highlights the importance of authentic leadership in reducing employee turnover intention by influencing employees’ perceived support of their supervisor. Our study contributes to the generalizability of the effects of authentic leadership on employee turnover intention across different research contexts. Given the importance of perceived support in organizational effectiveness, our study sheds new light on the role of PSS in the mechanism by which authentic leadership influences employee turnover intention. The moderated mediation model of PSS as well as organizational identification showed empirical evidence to clarify the complex relationship between authentic leadership and employee turnover intention. Our finding shows that with high level of organizational identification, the effect of PSS on turnover intention is stronger and PSS also shows the stronger mediating effect.

### Limitations and future research

Although this study is meaningful, it has some limitations. First, this study has common method bias because we only used self-reports of employees to measure all variables. To address this issue, we made every attempt to minimize concerns of common method variance before collecting data. For example, we introduced a time lag between the measurement of our independent variables and outcome variables. We also ensured the anonymity and confidentiality of the respondents. Furthermore, we counterbalanced the order of the questions. Lastly, we provided clear instructions for completing the measure, with definitions to avoid confusion (Podsakoff et al., 2003). In addition, it is worth noting that the use of self-reported data is generally considered to be the most valid approach when assessing perceptual outcomes and internal states, such as feelings and perceptions (Chan, 2009). Given that the main variables studied referring to the employees’ personal perceptions (e.g., PSS, organizational identification, and turnover intention), and employee-reported authentic leadership with Authentic Leadership Questionnaire (ALQ; Walumbwa et al., 2008) is the most frequently used (Gardner et al., 2011), we argue that using self-reported data in our study is not a major limitation. Future researchers may extend this research by using multi-source data.

Second, another limitation derives from our cross-sectional research design. Although the results confirm all of our hypotheses, one must be careful when interpreting the results regarding causality. For example, it seems plausible that employees with high PSS will assess organizations or bosses more favorably, subsequently causing the employee to evaluate the manager as more authentic. Future research using a longitudinal design would allow for more robust statements about the direction of causality and to capture the dynamic nature of leadership and employee work outcomes.

Third, we need to employ various research designs to investigate the effects of authentic leadership on employee turnover intention. Recently, turnover research has employed repeated measures designs. For example, Haider et al. (2020) showed how and why perceptions of organizational politics influence turnover intention across time with a three-wave study with six-month time lag among waves. Therefore, in future research, it might be necessary to track changes in employee turnover intentions over a specific period of time to investigate how changes in authentic leadership influence it.

Lastly, our research findings are mainly based on the data collected from organizations in South Korea, which might threaten the external validity so that we cannot generalize the findings of our study to a broader context. Future research could be done with data collected from diversified contexts.

## Data availability statement

The raw data supporting the conclusions of this article will be made available by the authors, without undue reservation.

## Ethics statement

Ethical review and approval was not required for the study on human participants in accordance with the local legislation and institutional requirements. The patients/participants provided their written informed consent to participate in this study.

## Author contributions

KJ conceived the study, developed the materials, and gathered data. YS conducted the analyzes. KJ and ZH prepared the draft manuscript. KJ, ZH, and YS revised the manuscript. All authors contributed to the article and approved the submitted version.

## Conflict of interest

The authors declare that the research was conducted in the absence of any commercial or financial relationships that could be construed as a potential conflict of interest.

## Publisher’s note

All claims expressed in this article are solely those of the authors and do not necessarily represent those of their affiliated organizations, or those of the publisher, the editors and the reviewers. Any product that may be evaluated in this article, or claim that may be made by its manufacturer, is not guaranteed or endorsed by the publisher.
